# Binding Mechanism and Electrochemical Properties of M13 Phage-Sulfur Composite

**DOI:** 10.1371/journal.pone.0082332

**Published:** 2013-11-26

**Authors:** Dexian Dong, Yongguang Zhang, Sanjana Sutaria, Aishuak Konarov, Pu Chen

**Affiliations:** Department of Chemical Engineering, University of Waterloo, Waterloo, Ontario, Canada; University of Houston, United States of America

## Abstract

Self-assembly of nanostructured materials has been proven a powerful technique in material design and synthesis. By phage display screening, M13 phage was found to strongly bind sulfur particles. Fourier transform infrared and X-ray photoelectron spectroscopy measurements indicated that the strong sulfur-binding ability of M13 phage derives from newly generated S-O and C-S bonds. Using this phage assembled sulfur composite in a lithium battery, the first discharge capacity reached 1117 mAh g^-1^, which is more than twice that of the sulfur only cathode. Besides, the negative polysulfide shuttle effect in a lithium-sulfur battery was significantly suppressed.

## Introduction

Self-assembly of nanostructured materials allows material design with hierarchical order and has been proven a powerful technique in material synthesis [1,2]. Viruses have the inherent capability of self-assembly and could be used for fabricating the nanostructure [3]. M13 phage is filamentous (880 nm long and 8 nm wide) and harmless to humans [4]. Angela M. Belcher’s lab at MIT has extensively experimented with inorganic material templating using M13 phage mutant E4 for different applications [5-7], including templating Co_3_O_4_ [8] and FePO_4_ [9] for lithium ion batteries. The templating mechanism of E4 virus was attributed to the interaction of cations with the carboxylic acid groups of tetraglutamate [9]. 

Sulfur has recently received substantial interest as one of the most promising cathode materials for lithium rechargeable batteries because it is inexpensive, environmentally benign, has a high theoretical specific capacity of 1672 mAh/g and theoretical energy density of 2600 Wh/kg. [10-12] However, the development of Li/S batteries has encountered several challenges from capacity loss to short cycle life [13]. Practical application of sulfur as a cathode active material in lithium rechargeable batteries has not yet been successful. The main reasons for these problems are the low electronic conductivity of sulfur, polysulfide dissolution, and the shuttle effect [14-16]. During the discharge phase, molecules of elemental sulfur, S8, accept electrons, generating lithium polysulfides (Li_2_S_n_, 2 <n<8), which dissolve into the liquid electrolyte. As the discharge process continues, some lithium polysulfide will diffuse to the lithium anode and react with the lithium metal to regenerate the lower order polysulfides. These species diffuse back to the ‘sulfur’ cathode to generate higher forms of polysulfide again, thus creating a shuttle mechanism [17]. As the cycling continues, more and more lithium polysulfide diffusion occurs. During the charge phase, more energy is needed to force the lithium polysulfide back to the cathode, which causes a higher charge capacity. 

Although sulfur is elemental and not ionic, we believe M13 phage can be used to template sulfur. Our initial research plan was to first use phage display technology to determine the specific polypeptide sequence that could bind to sulfur, and then clone this sequence into the major coat protein p8 of M13 phage to enable the expression of 2700 polypeptides on the surface [18]. Finally, use this M13 mutant to template sulfur and make the virus-assembled lithium-sulfur composite. Through phage display screening, we found that the major coat protein p8 of M13 phage can naturally and strongly bind to elemental sulfur. Phage cloning is therefore unnecessary. The binding mechanism of phage to elemental sulfur is further investigated by combining Fourier transform infrared and X-ray photoelectron spectroscopy analyses. Battery tests showed that phage templating enhanced the discharge capacity of the lithium-sulfur composite and effectively reduced the polysulfide shuttle effect. Herein, we report our results.

## Results

### 1: M13 phage naturally strongly binds sulfur particles

**Figure 1:**
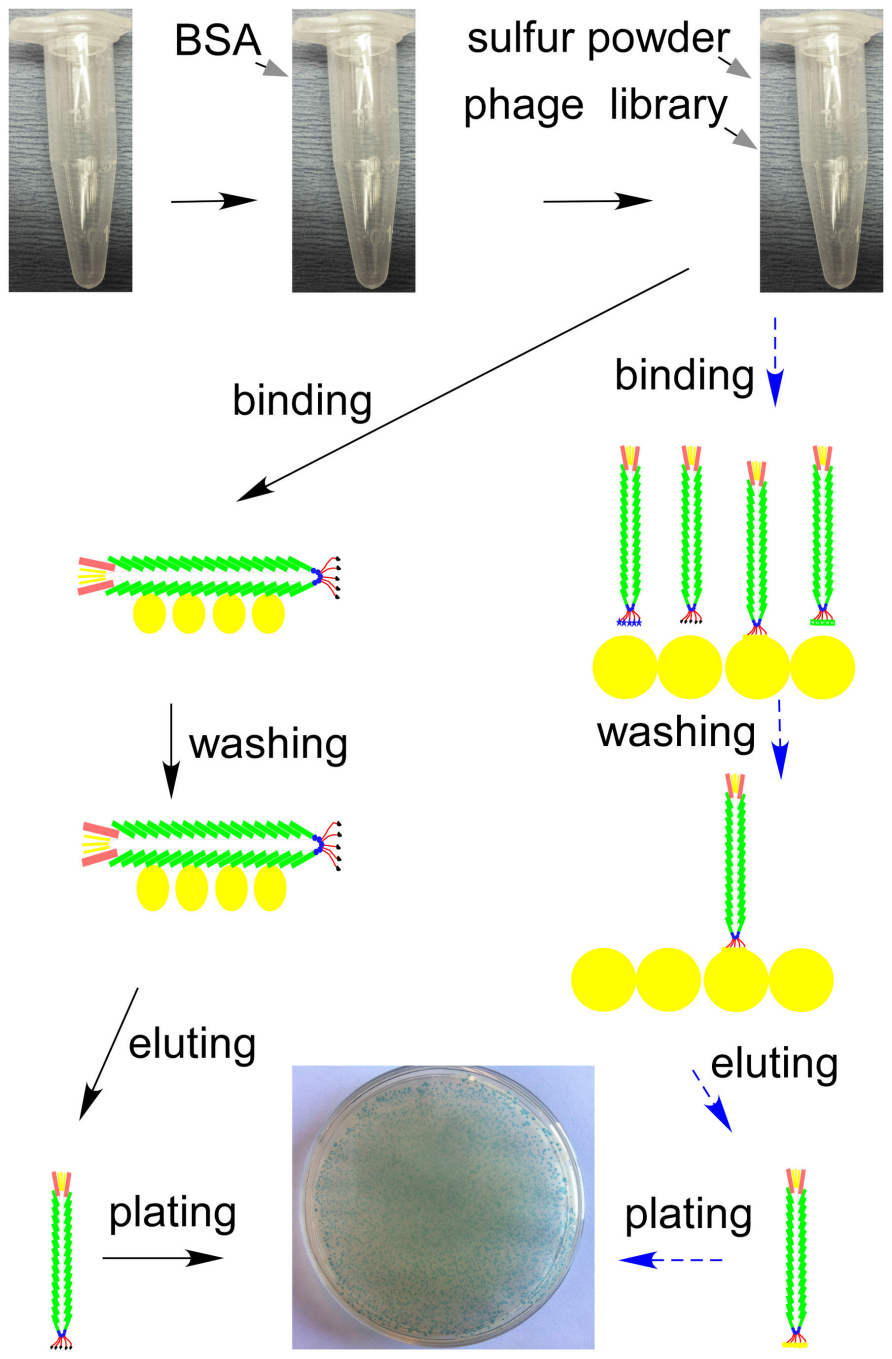
Graphical illustration for the sulfur-binding phage display screening. Blue dashed arrow: the expected pathway; Black solid arrow: the observed pathway.

By serial phage display screening, we expected to identify the special p3 sequence in the phage display peptide library that could bind to sulfur in the way shown in Figure 1 (in the blue dashed arrow direction). Initially, all the plates for 0.1 % up to 0.9 % Tween 20, in 0.1 % gradients, showed blue plaques and indicated the presence of M13 phage-sulfur binding. A representative plate with blue plaques is shown in the bottom middle in Figure 1. All the batch screenings from 1.0 % up to 30 % Tween 20, in 0.2 % or 1 % gradients, showed blue plaques and indicated that M13 phage-sulfur binding was very strong and that the phage is not lost by repeated washing. The highest concentration for washing was 30 % Tween 20, because higher concentrations are too viscous to separate the sulfur particles by centrifuging. We assumed that washing 10 times may not be enough, so we tested 6 %, 12 %, 20 %, and 30 % Tween 20 and washed 20 times. In this scenario, the unbounded phage could be diluted to 1/20^20^=1/1.05 x 10^26^ times and could be negligible. These tests showed the same results as those washed 10 times. Thus, washing 10 times is enough. M13 phages bind strongly to sulfur particles.

### 2: The binding site of M13 phage with sulfur could be the surface area of P8 coat protein

There are four possible mechanisms for binding: (a) sulfur binding to the ends of phage (blue dashed arrow pathway in Figure 1); (b) sulfur binding to the side of the phage (black solid arrow pathway in Figure 1); (c) phage penetration into the sulfur particles; and (d) phage binding onto the surface of the microfuge tube. The possibility for binding onto the surface of a microfuge tube was eliminated by using BSA blocking buffer. Phage penetration into the sulfur particles is unlikely. The interior molecular force of sulfur is strong, and we never observed this phenomenon during subsequent transmission electron microscopy. To confirm that M13 phage-sulfur binding resulted from either end binding or side binding, we randomly picked 20 plaques from the solid plates of the original Ph.D. ™-7 libraries, amplified them individually and used these pure amplified clones to do the 23 % Tween 20 washing. All twenty pure M13 phage clones showed blue plaques on the plates. This indicates that phages with different specific p3 sequences but the same p8 sequence can bind strongly to sulfur. Thus, we concluded that the binding site was along the side of the phage (protein P8) and not at the ends where the protein P3 is located. This means that the surface peptide sequence of major coat protein P8 is able to naturally and strongly bind to sulfur particles, and our previous molecular cloning plan is unnecessary. 

### 3: Transmission electron microscopy (TEM) confirmation

**Figure 2:**
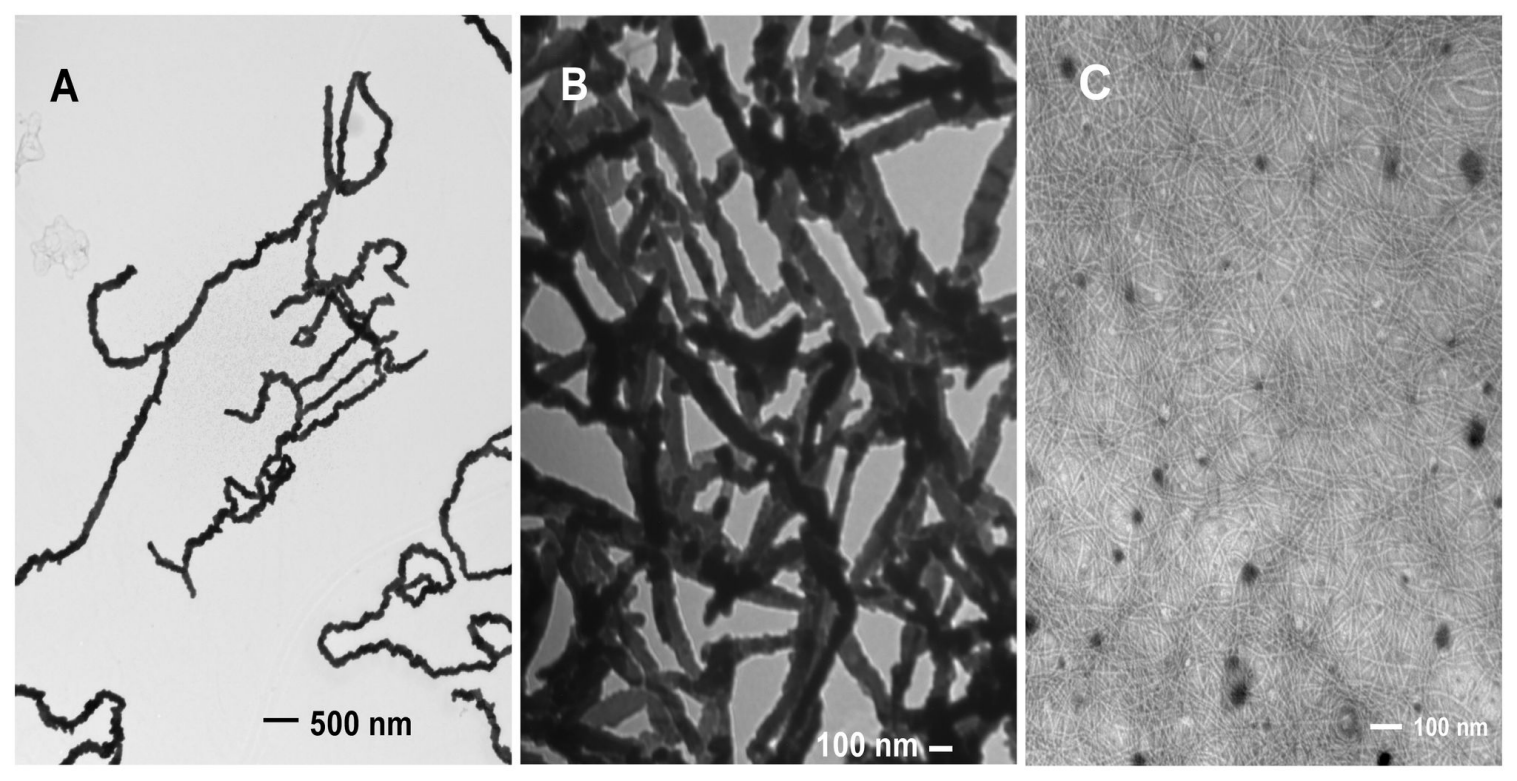
TEM images for the structures of phage covered by the sulfur nanoparticle (size approximately 47 nm). A: magnification 13500×; B: magnification 46000×; C: magnification 64000× and negative staining with 0.5 % phosphotungstic acid.

To confirm our deduction, TEM tests were conducted, and the images are shown in Figure 2. The sulfur nanoparticles bound to the side of the phage, and as a result, formed a beaded chain structure. Figure 2A shows the separated structure of phage covered by the sulfur nanoparticle (size approximately 47 nm) under direct magnification 13500×. Figure 2B shows a more dense structure of phage covered by the sulfur nanoparticle under direct magnification 46000×. To show the phage presence in these structures, negative staining was performed. Figure 2C shows the phage structure treated with 0.5 % phosphotungstic acid for 10 seconds under direct magnification 64000×. The sulfur nanoparticle was unbound by phosphotungstic acid [19], and the phages linked end to end and intertwined. The pure phage without sulfur, treated with 0.5 % phosphotungstic acid, also shows the linkage structure similar to that in Figure 2C. The reason for the phage linkage is unclear, but it proves that the sulfur nanoparticles do bind to M13 phage and that the binding site of sulfur-phage is along the side of the phage---the surface area of major coat protein P8. 

### 4: Fourier transform infrared spectroscopy

**Figure 3:**
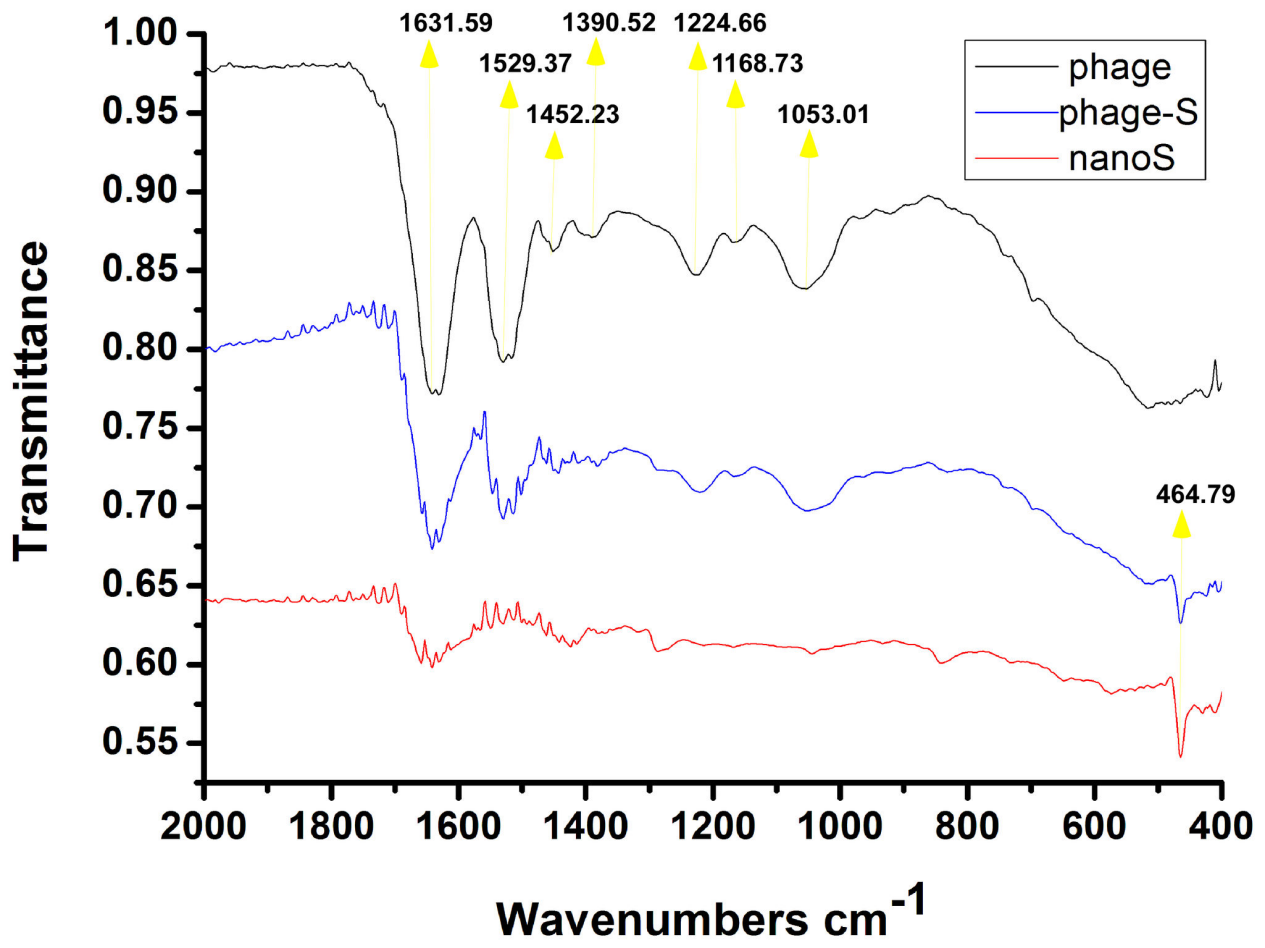
Fourier transform infrared spectra of phage, sulfur nanoparticle and phage-sulfur composite.

The Fourier transform infrared (FTIR) spectra of the nanoS, phage and phage-S composite are given in Figure 3. Two main bands are observed in the spectral region between 1500 and 1800 cm^-1^ that can be identified as the amide I band, attributed to the amide plane C=O stretching vibration [20], centered at 1631.59 cm^-1^ and the amide II band, associated with the amide plane N-H bending and C–N stretching, located at 1529.37 cm^-1^. A band at 1452.23 cm^-1^ is due to CH_2_ scissoring, CH_2_ bending and asymmetric CH_3_ bending, and another band at 1390.52 cm^-1^ is due to symmetric CH_3_ bending [21,22]. The band at 1224.66 cm^-1^ is due to the amide III, C-N stretching and N-H in plane bending, often with significant contributions from CH_2_ wagging vibrations [20]. Another band at 1168.73 cm^-1^ is due to C-C and C-OH stretching [20]. The band at 1053.01 cm^-1^ is due to O–C=O stretching [20-22]. The FTIR spectrum of the nanoS shows a weak band at 464.79 cm^-1^, which could be attributed to the S-S stretching [23]. Other weak bands may have originated from some unknown component residues of the sulfur nanoparticle water dispersion. 

The FTIR spectrum of the phage-S composite shows combinational characteristic bands of pure phage together with nanoS. This combinational FTIR spectrum indicates that the sulfur has been intercalated into the M13 phage [24]. The bands for CH_2_ wagging, C-C stretching, C-OH stretching and O–C=O stretching are slightly shifted in position to 1220.80, 1166.80 and 1051.08 cm^-1^, respectively. These downshifts suggest that the side-chains of the AEGDD (the surface amino acid sequence of the phage major coat protein P8), the COOH groups of glutamate (E) and aspartate (D), and the C-C groups of alanine (A), glutamate and aspartate, have some interaction with sulfur, possibly through S-O or C-S bonds. 

### 5: X-ray Photoelectron Spectroscopy (XPS)

M13 phage is mainly composed of 2700 copies of major coat protein P8. The P8 amino acid sequence is: AEGDDPAKAAFNSLQASATEYIGYAWAMVVVIVGATIGIKLFKKFTSKAS. AEGDDPAK is the surface amino acid of the M13 phage [25]. Based on this knowledge, the carbon chemical state can be classified as: amide C=O, -CH_3_, -CH_2_-, O-C=O, -CH-, -C-OH and -C_6_H_6_; The oxygen chemical states are C=O, O-C=O, and -C-OH; Nitrogen chemical states are: amide plane –NH- and -NH_2_. The sulfur composition is small, because only a single methionine (-CH_2_-S-CH_3_) group is contained in protein P8, and there is no cysteine. 

**Figure 4:**
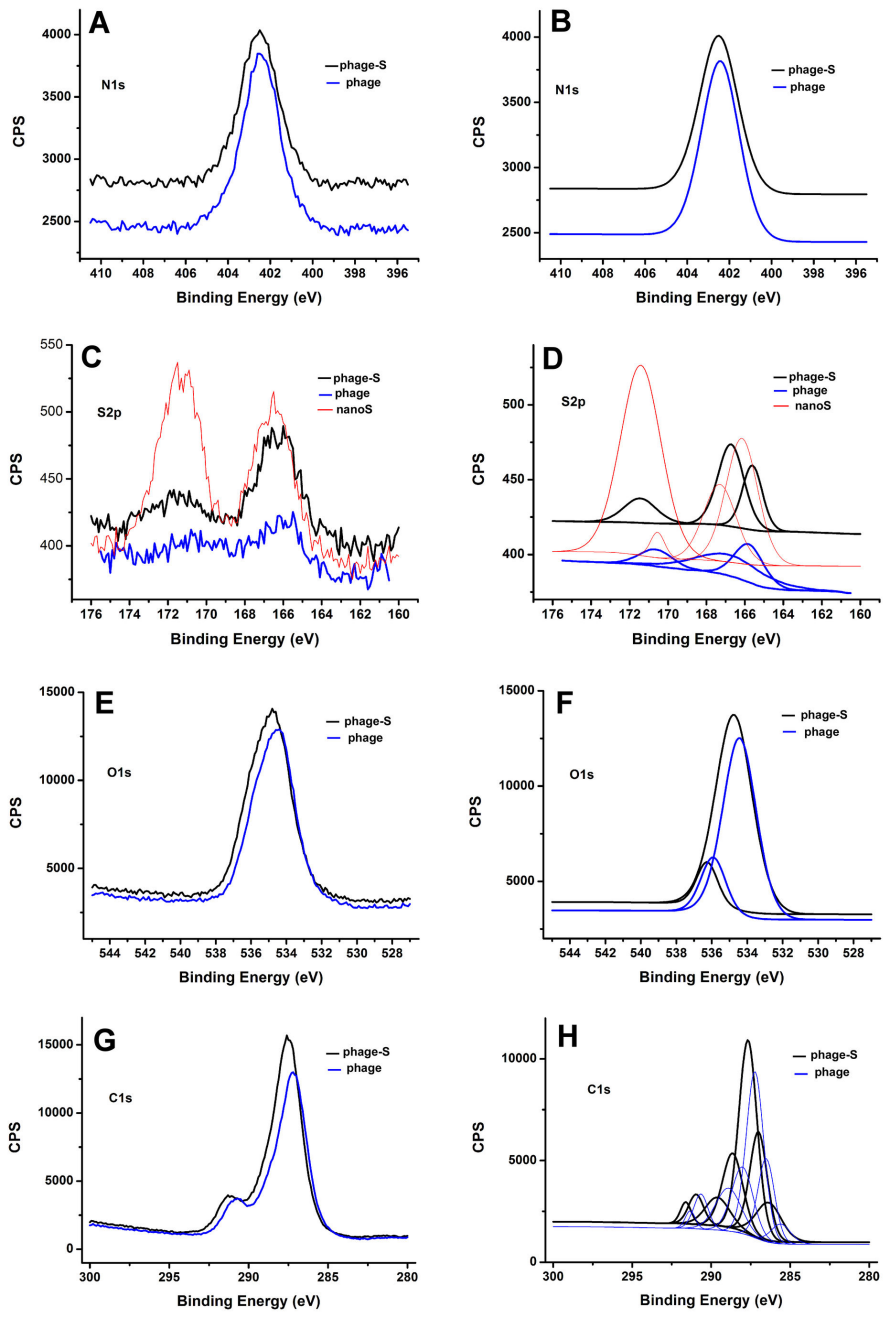
Origin spectra of N1s (A), S2p (C), O1s (E) and C1s (G) regions by X-ray Photoelectron Spectroscopy and deconvolution spectra of N1s (B), S2p (D), O1s (F) and C1s (H) for phage (blue), phage-sulfur composite (black) and sulfur nanoparticle (red).

Sulfur interaction with phage was examined by investigating the sulfur 2p (S2p), oxygen 1s (O1s), carbon 1s (C1s) and nitrogen 1s (N1s) spectra. Figure 4A and 4B show the original spectra and the deconvoluted spectra of the N1s region of the XPS for the phage (blue) and phage-S composite (black). Both materials have one peak (402.5 eV), which could be attributed to amide plane –NH- group in the peptide chain and the -NH_2_ group of lysine, whose location has no shift. This indicates that nanoS particles do not interact with these N groups, and hence, the background of the XPS equipment has no shift difference. 

Figure 4C and 4D show the original spectra and deconvoluted spectra of the S2p region of the XPS for phage (blue), phage-S composite (black) and nanoS (red). The nanoS shows two peaks that reflect the non-oxidized (166.5 eV) and oxidized (171.5 eV) forms of sulfur [26]. Pure phage has one small peak (166.1 eV), which could be attributed to the C-S-C group of methionine. In the S2p spectrum of the phage-S composite, the peak attributed to the oxidized (171.5 eV) form of sulfur shows a significant drop in number of electrons, and another peak (166.6 eV) shows a small change in the number of electrons. From further deconvolution analysis (see Figure 4D), the spectra of three samples could be deconvoluted as three main peaks, except nanoS, which has a small fourth peak, although S2p should be deconvoluted to S2p3/2 and S2p1/2 [27]. Compared to the peaks of the phage (165.9, 167.3, and 170.8 eV), two peaks of the phage-S composite (165.6 and 166.7 eV) have 0.3 eV and 0.6 eV downshifts, but another peak (171.5 eV) has a 0.7 eV upshift. Compared to the peaks of nanoS (166.2, 167.3 and 171.5 eV), two peaks of the phage-S composite (165.6 and 166.7 eV) have 0.6 eV and 0.6 eV downshifts, but another peak (171.5 eV) has no shift. This deconvolution analysis demonstrates that when nanoS met the phage, the oxidized form of sulfur gained electrons, most likely from the O and C atom, and transformed to the non-oxidized form of sulfur. The peak shifts indicate that the C-S-C group is not the only factor; there may also be S-O bonds present. 

Figure 4E and 4F show the original spectra and deconvoluted spectra of the O1s region of the XPS spectra for phage (blue) and phage-S composite (black). There are only two peaks by the deconvolution analysis, which could be assigned to the C=O (535.9 eV) and O-C=O (534.4 eV) groups. The peak (534.8 eV) for the phage-S composite upshifts 0.4 eV towards higher binding energy compared with the peak (534.4 eV) for the phage. This upshift confirms that the O atom from the amide C=O and O-C=O groups donates electrons to the S atom and becomes more oxidized. 

Figure 4G and 4H show the original spectra and deconvoluted spectra of the C1s region of the XPS spectra for phage and phage-S composite. Although the seven peaks deconvolution analysis is most likely inaccurate, the peak (287.6 eV) for the phage-S composite shown in Figure 4G upshifts 0.4 eV towards higher binding energy compared with the peak (287.2 eV) for the phage. All seven deconvolution peaks of the phage-S composite shown in Figure 4H upshift towards higher binding energy when compared with the phage peaks. These upshifts confirm that the C atom also donates electrons to the S atom.

Combining the FTIR and XPS analyses, we could conclude that the O and C atom from the surface of the phage, especially the O-C=O groups of glutamate and aspartate, donate electrons to sulfur, resulting in the generation of S-O and C-S bonds. Sulfur binds to the O-C=O groups of glutamate and aspartate, and the amide C=O groups of the peptide chain by S-O and C-S bonds.

### 6: Electrochemical performance of the phage-sulfur composite

**Figure 5:**
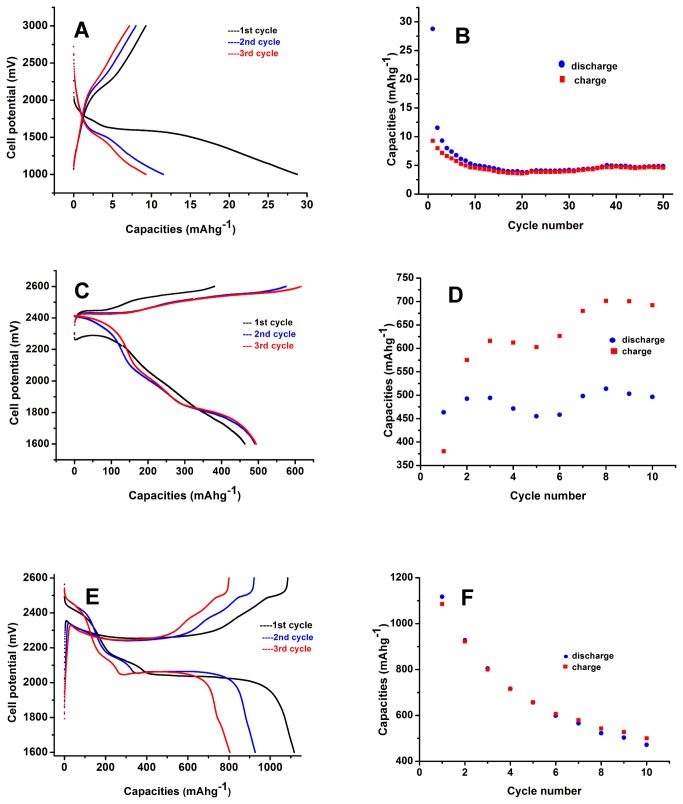
Charge–discharge curves of phage (A), sulfur nanoparticle (C) and phage-sulfur composite (E) and cycle performances of phage (B), sulfur nanoparticle (D) and phage-sulfur composite (F) at 0.1 C.

After the pH of the phage solution was adjusted to 7.0 with 2 M LiOH and dried at 70°C in an oven, pure phage material was mixed with 10 wt% PVdF and 10 wt% Super P^®^ Li carbon black. This slurry is used as the cathode material, and lithium foil is applied as the anode. The resulting data (shown in Figure 5A and 5B) indicated that pure phage has no or little contribution on the discharge capacity and cyclability. The discharge capacity at the first cycle is only 29 mAh g^-1^ and it most likely resulted from the Super P^®^ Li carbon black.

After the nanoS water dispersion was dried at 70°C in an oven, the nanoS powder was used as the control to make the battery. The capacity curve in Figure 5C shows that the nanoS battery had the typical discharge plateau (approximately 1.9-2.1 V) of sulfur [16]. Its discharge capacity is approximately 500 mAh g^-1^ (494 at 3^rd^ cycle). Its charge capacity increases quickly and significantly (see Figure 5C and 5D) (380 mAh g^-1^ at 1^st^ cycle, 616 mAh g^-1^ at 3^rd^ cycle and 692 mAh g^-1^ at the 10^th^ cycle). As previously reported, the main reason for this phenomenon is the shuttle effect [14-16].

The initial profiles of the galvanostatic charge–discharge tests of the phage-S cathode are shown in Figure 5E. Three main plateaus appear in the potential profiles, which could be attributed to three main electrochemical reactions taking place at the sulfur cathode [15]. The first electrochemical reaction is presented by a short discharge plateau at approximately 2.45 V and is related to the formation of the higher-order lithium polysulfides (Li_2_S_n_, n≥5). The second plateau, approximately 2.15 V, in the discharge profiles reflects the following electrochemical transition of the polysulfide to the lithium sulfide Li_2_S_4_. The third plateau is approximately 2.05 V in the discharge profiles and reflects the electrochemical transition of the polysulfide to short polysulfide species, such as Li_2_S_3_, Li_2_S_2_, and Li_2_S. Whereas the 2.05 V discharge plateaus had no significant difference between the first and third cycles, the higher voltage plateaus diminish and almost disappear after a few cycles. This could be due to the activation of the phage-S cathode upon the initial cycles and achieving a steady state for the polysulfide formation. The system’s discharge capacity mainly depends on the 2.05 V plateau.

The phage-S cathode performance (shown in Figure 5E and 5F) was enhanced compared with that of the nanoS cathode as shown in Figure 5C and 5D. The discharge capacity at the first cycle is 1117 mAh g^-1^, which is more than twice that of the nanoS cathode. The difference between the discharge capacity and the charge capacity at the same cycle is minimal (see Figure 5E and 5F), which indicates the negative shuttle effect of polysulfides has been drastically suppressed. Our repeat test data (at least 8 repeats) showed similar results that the phage assembly could raise the discharge capacity within 10 cycles and drastically suppress the negative shuttle effect. However, the phage assembly could not improve the sulfur cyclability (see Figure 5F). High initial capacity dropped rapidly. After the 10^th^ cycle, the discharge capacity decreased to 471 mAh g^-1^. 

## Discussion

Based on our finding that the sulfur nanoparticles could strongly bind to the side of the phage and form a beaded chain structure, the discharge capacity (1117 mAh g^-1^) enhancement of the phage-S cathode within 10 cycles could partly be attributed to the templating effect of the phage. The sulfur nanoparticles were evenly distributed and bound over the surface of the nanowired phage and formed a fine structure that increased the area of sulfur in contact with the conductive material. This increase of sulfur contact area will improve sulfur efficiency and present the increase of discharge capacity. Besides, comparing the discharge curves of the nanoS cathode in Figure 5C and phage-S cathode in Figure 5E, the third (2.05 V) discharge plateaus of phage-S cathode is much wider than that of nanoS cathode without phage. It could be interpreted as that phage could bind the high order polysulfides. After elemental sulfur accepts electrons and becomes high order polysulfide, these polysulfides, like elemental sulfur, still bind strongly to the phage surface instead of diffusing out of the cathode. For the nanoS cathode without phage binding, the polysulfides dissolve into electrolyte and diffuse out of cathode quickly. This polysulfide-binding effect of phage contribute another partial increase of discharge capacity. 

Since the main reason for shuttle effect is the polysulfide diffusing out of the cathode, and in our phage-S cathode, the high order polysulfide still bind strongly to the phage surface instead of diffusing out of the cathode, the shuttle effect should be effectively suppressed. 

The discharge capacity of the nanoS cathode without phage maintain at approximately 500 mAh g^-1^. It could be attribute to that all the polysulfides be pushed back to the cathode during charge phase. For the the phage-S cathode, the reason for discharge capacity dropping maybe is that the newly generated S-O and C-S bonds between phage and sulfur become weak as lithium ion joining in the polysulfide complex during late discharge phase, Parts of low order polysulfide detach from the phage surface and diffuse out of cathode. During charge phase, the low order polysulfide cannot be pushed back to the cathode because of the weak binding ability of phage and the spatial blockade of phage.

For further industrial application, both M13 phage and sulfur nanoparticles require scaling up of production. According to our research [28], scaling up phage production is possible. Currently, we are able to attain a phage yield of 0.25 g dry weight L^-1^ LB culture medium, on a 200 mL culture scale. Upgrading to a 1L culture scale has proven feasible. The cost for phage production is inexpensive; the only required steps are to ferment *E. coli* and inoculate phage. Harvesting phage requires the addition of only small amounts of acid and/or alkaline. Elemental sulfur has high abundance in the earth's crust. The sulfur nanoparticle product we used is cheap, which means that large scale production of sulfur nanoparticles is economically possible. Additionally, M13 phage only infects bacteria and is essentially harmless to the human body. Thus, exploitation of M13 phage for large scale production does not pose a safety threat. After battery assembly and electrochemical testing, we re-opened the battery cells to check the phage infectivity (infectivity test is same as the presence verification of eluted phages mentioned in the methods section). At this point, the phage had lost infectivity to *E. coli*. This proves that it is safe for disposal as it will not impact the environment. Hence, there is great potential for the industrialization of this phage-based lithium-sulfur battery.

According to our results, M13 phage assembly does not improve the cycle life of lithium-sulfur batteries. Low order polysulfides still detach from the phage surface and diffuse out of cathode. There are several published papers regarding cycle life improvement of the lithium-sulfur battery [29-32]. A microporous carbon paper [29] was applied to localize the soluble polysulphide species near the cathode. To limit polysulphide dissolution, sulfur particles were encapsulated by TiO2 shell [31], or constrained into the interwoven channels of mesoporous carbon CMK-3 [32]. Based on these design, choosing a conductive material to cover (or wrap) the phage-S composite may prevent the discharge capacity from fading. We tried to use polypyrrole, but a solvent to dissolve polypyrrole must first be found.

For the phage binding mechanism, one assumption is that there is an interaction of cations with the carboxylic acid groups of glutamate and aspartate. According to our experiments, phage has the ability to selectively bind or absorb some elemental substances or oxide compounds, such as sulfur, silicon, platinum, zinc and titanium oxide. Revealing the phage binding mechanism requires further cumulative data.

## Materials and Methods

### 1: Phage display screening

M13 phage is composed of one circular single-stranded DNA and is encapsulated in approximately 2700 copies of major coat protein p8 and capped with 5 copies of minor coat protein p3 [18]. The Ph.D. ™-7 phage display peptide library was bought from New England Biolabs Inc., Canada. It is a combinatorial library of 10^9^ electroporated sequences fused to the minor coat protein p3. Library screening was conducted according to the manual instructions with some revisions (http://www.neb.com/nebecomm/products/productE8102.asp). Microfuge tubes (1.5 mL) were coated with BSA (bovine serum albumin) by using blocking buffer (0.1 M NaHCO_3_, pH 8.6, 5 g L^-1^ BSA, stored at 4 °C). Each tube was filled completely with blocking buffer and incubated for 4 hours at 4 °C. The blocking buffer was then discarded and the coated tubes were washed rapidly 6 times with TBST (50 mM Tris-HCl, 150 mM NaCl, 0.1 % (v/v) Tween-20, pH 7.5). The washing was performed quickly to avoid drying out of the tubes (see Figure 1).

Sulfur (0.05 g) (Sigma, Canada) was suspended in 1.0 mL TBST and sonicated for 1 hour. The mixture was transferred to the microfuge tube coated with BSA. Ten microliters of phage library solution (phages of 1.5×10^10^ with 2.0×10^9^ different sequences) was added to each tube and incubated for one hour with gentle shaking. Each tube was rinsed with 1.0 mL TBST ten times to wash off unbound phage (mixed by vortex and centrifuged for 1 min at 10,000 rpm). Bound phages were eluted by incubating with 1.0 mL 0.2 M Glycine-HCl, pH 2.2, for eight minutes and carefully transferring into a new 1.5 mL microfuge tube. The solution was immediately neutralized with 150 μL of 1 M Tris-HCl, pH 9.3. The presence of eluted phages was verified with agar solid plates containing 50 mg L^-1^ X-gal (5-bromo-4-chloro-indolyl-β-D-galactopyranoside) and 40 mg L^-1^ IPTG (isopropylthio-β-galactoside). The same procedure was repeated with increasing Tween 20 concentrations.

### 2: Transmission electron microscopy (TEM)

For preparation of phage-sulfur composite (phage-S), 10 μL of 50 ppm sulfur nanoparticle (nanoS) water dispersion (US Research Nanomaterials, Inc.) was mixed with 10 μL phage solution (1.5×10^11^ plaque forming units (pfu) mL^-1^) and was incubated at room temperature for 10 min. Ten microliters hybrid solution was applied to a formvar-coated TEM grid for 3 min. The solution was wicked from the edge of the grid by a wedge of wipe paper. When negative staining [19] was needed, 10 μL of 0.5 % phosphotungstic acid (PTA, pH 5-7) was applied on the sample for 10 seconds. The staining solution was immediately wicked from the edge of the grid by a wedge of wipe paper. The TEM grids were dried by pressurized air, kept at room temperature and characterized under a transmission electron microscope (Philips CM10). 

### 3: M13 phage production

*E. coli* ER2738 was cultured with LB Medium (10 g L^-1^ tryptone, 5 g L^-1^ yeast extract and 5 g L^-1^ NaCl, pH 7.0-7.5) for 16 hours at 37 °C and 220 rpm. Two hundred milliliters culture in a 500 ml Erlenmeyer flask was inoculated with 200 μL *E. coli* culture storage and 10 μL phage solution (1.5×10^11^ pfu mL^-1^, resulting from one single pure clone). After culturing, the bacterial cells were removed by centrifugation at 5000 rpm for 10 min. The supernatant was then transferred to fresh centrifuge tubes and a repeat centrifugation, to clean up the bacteria residue, was performed at 12,000 rpm for 10 min. The pH of the resultant supernatant was adjusted to pH 4.2 with 5 M HCl. The phage pellet was harvested by centrifugation at 13,000 rpm for 10 min at room temperature. To remove the LB medium residue in the collected phage, the pellets were resuspended in 40 mL Milli-Q water by vortexing and were centrifuged at 13000 rpm for 10 min at 20 °C. Ten milliliters Milli-Q water was added to the pellet, and the pH was adjusted to 7.0 with 2 M LiOH. The phage was resuspended by vortexing and was stored at 4 °C for further use.

### 4: Fourier transform infrared and X-ray Photoelectron Spectroscopy

Fourier transform infrared (FTIR) spectra between 2,000 cm^-1^ and 400 cm^-1^ were recorded with a Bruker Vertex 70 with a platinum ATR-QL diamond accessory, a resolution of 4 cm^-1^ and 16 scans. X-ray Photoelectron Spectroscopy (XPS) was performed using a multi-technique ultra-high vacuum Imaging XPS Microprobe system (Thermo VG Scientific ESCALAB 250) equipped with a hemispherical analyzer (of 150 mm mean radius) and a twin anode non-monochromatic Al Kα source. The analysis chamber was maintained at 2×10^-10^ mbar. A combination of low energy electrons with an emission current of 0.2 mA was used for the charge compensation on the non-conducting samples during the analysis. Deconvolution spectra were produced by the CasaXPS2316PR1 software (www.casaxps.com).

### 5: Preparation of the phage-sulfur (phage-S) composite

Each tube contained 10 mL M13 phage suspension resulting from the 200 mL culture medium. Four hundred microliters of 10 wt% nanoS water dispersion (US Research Nanomaterials, Inc.) were added to each tube. After mixing well by vortex and shaking for 2 hours, the tube was covered by Bio-Shield® wrap (Fisher Scientific, Canada), and the phage-S suspension was dried in a regular oven at 70 °C for almost two days.

### 6: Electrochemical measurements

The electrochemical performance was investigated using coin-type cells (CR2025). Lithium metal foil was used as the anode. The liquid electrolyte was 1 M LiCF_3_SO_3_ (lithium bistrifluoromethanesulfonamide, Sigma, 96 % purity) in TEGDME (tetraethylene glycol dimethyl ether, Sigma, 99 % purity) and DME (dimethoxy ethane) (3:1 by volume). The separator was microporous polypropylene (Celgard 2400). The composite cathode was prepared by mixing 80 wt% phage-S composite, 10 wt% polyvinylidene fluoride (PVdF, Kynar, HSV900) as a binder and 10 wt% Super P^®^ Li carbon black (MTI, 99.5 % purity). N-methyl-2-pyrrolidinone was used as a dispersant (NMP, Sigma, 99.5 % purity). The resultant slurry in NMP was spread onto a circular piece of nickel foam (MTI, 99 % purity) with a 1 cm diameter. After drying at 60 °C in a vacuum oven for 5 h, the cathode was pressed at 8 MPa by a hydraulic press to achieve solid contact between the active material and nickel foam. The phage-S composite loading on each cathode was approximately 2 mg cm^-2^. The coin cells were assembled in a Braun glove box filled with high purity argon (99.9995 % purity). The cells were tested galvanostatically on a multichannel battery tester (BT-2000, Arbin Instruments) between 1.6 and 2.6 V vs Li+/Li anode at a constant current density of 0.1 C-rate. The applied currents and specific capacities were calculated on the basis of the weight of sulfur in the cathode. The sulfur content in the phage-S composite was determined using a CHNS elemental analyzer (Elementar Vario Micro Cube, Elementar, Germany).

## Conclusions

By phage display screening, we found that M13 phage could bind sulfur particles strongly, and the binding site was on the sides of the phage, not the ends of the phage. Specifically, the binding was with the major coat protein P8. By combining the Fourier transform infrared and X-ray photoelectron spectroscopy analyses, we deduced that sulfur bound to the O-C=O groups of glutamate and aspartate and/or the amide C=O groups of the peptide chain via newly generated S-O and C-S bonds. Using this phage-sulfur material as a cathode and lithium as an anode, the discharge capacity at the first cycle reached 1117 mAhg^-1^, which is more than twice that of a sulfur particle cathode. The polysulfide shuttle effect was significantly suppressed by the phage binding sulfur cathode. 

## References

[1] Nie Z, Petukhova A, Kumacheva E (2010) Properties and emerging applications of self-assembled structures made from inorganic nanoparticles. Nat Nanotechnol 5: 15-25. doi:10.1038/nnano.2009.453. PubMed: 20032986. 20032986

[2] Yan Y, Lin YY, Qiao Y, Huang JB (2011) Construction and application of tunable one-dimensional soft supramolecular assemblies. Soft Matter 7: 6385- 6398. doi:10.1039/c1sm05030c.

[3] Liu Z, Qiao J, Niu ZW, Wang Q (2012) Natural supramolecular building blocks: from virus coat proteins to viral nanoparticles. Chem Soc Rev 41: 6178- 6194. doi:10.1039/c2cs35108k. PubMed: 22880206. 22880206

[4] Hajitou A, Trepel M, Lilley CE, Soghomonyan S, Alauddin MM et al. (2006) A hybrid vector for ligand-directed tumor targeting and molecular imaging. Cell 125: 385-398. doi:10.1016/j.cell.2006.02.042. PubMed: 16630824. 16630824

[5] Nuraje N, Dang X, Qi J, Allen MA, Belcher AM (2012) Biotemplated synthesis of perovskite nanomaterials for solar energy conversion. Adv Mater, 24: 2885-2889. doi:10.1002/adma.201200114. PubMed: 22517374. 22517374

[6] Dang X, Yi H, Ham MH, Qi J, Belcher AM et al. (2011) Virus-templated self-assembled single-walled carbon nanotubes for highly efficient electron collection in photovoltaic devices. Nat Nanotechnol 6: 377-384. doi:10.1038/nnano.2011.50. PubMed: 21516089. 21516089

[7] Nam YS, Magyar AP, Lee D, Kim JW, Belcher AM et al. (2010) Biologically templated photocatalytic nanostructures for sustained light-driven water oxidation. Nat Nanotechnol 5: 340-344. doi:10.1038/nnano.2010.57. PubMed: 20383127. 20383127

[8] Nam KT, Kim DW, Yoo PJ, Chiang CY, Belcher AM et al. (2006) Virus enabled synthesis and assembly of nanowires for lithium ion battery electrodes. Science 312: 885-888. doi:10.1126/science.1122716. PubMed: 16601154. 16601154

[9] Lee YJ, Yi H, Kim WJ, Kang K, Belcher AM et al. (2009) Fabricating Genetically engineered high-power lithium ion batteries using multiple virus genes. Science 324: 1051-1055. PubMed: 19342549. 19342549 10.1126/science.1171541

[10] Cheng F, Liang J, Tao Z, Chen J (2011) Functional materials for rechargeable batteries. Adv Mater 23: 1695-1715. doi:10.1002/adma.201003587. PubMed: 21394791. 21394791

[11] Cairns EJ, Albertus P (2010) Batteries for electric and hybrid-electric vehicles. Annu Rev Chem Biomol Eng 1: 299-320 10.1146/annurev-chembioeng-073009-100942 22432583

[12] Yang Y, McDowell MT, Jackson A, Cha JJ, Cui Y et al. (2010) New nanostructured Li2S/silicon rechargeable battery with high specific energy. Nano Lett 10: 1486-1491. doi:10.1021/nl100504q. PubMed: 20184382. 20184382

[13] Zhang Y, Zhao Y, Sun KE, Chen P. (2011) Development in lithium/sulfur secondary batteries, Open Mater Sci J 5: 215-221

[14] Mikhaylik YV, Akridge JR (2004) Polysulfide shuttle study in the Li/S battery system. J Electrochem Soc 151: A1969-A1976. doi:10.1149/1.1806394.

[15] Barchasz C, Molton F, Duboc C, Leprêtre J, Alloin F et al. (2012) Lithium/sulfur cell discharge mechanism: an original approach for intermediate species identification. Anal Chem 84: 3973-3980. doi:10.1021/ac2032244. PubMed: 22482872. 22482872

[16] Manthiram A, Fu Y, Su YS (2012) Challenges and prospects of lithium-sulfur batteries. Acc Chem Res: ([MedlinePgn:]) doi:10.1021/ar300179v. PubMed: 23095063. 23095063

[17] Akridge JR, Mikhaylik YV, White N (2004) Li/S fundamental chemistry and application to high-performance rechargeable batteries. Solid State Ionics 175: 243-245. doi:10.1016/j.ssi.2004.07.070.

[18] Smith GP, Petrenko VA (1997) Phage display. Chem Rev 97: 391-410. doi:10.1021/cr960065d. PubMed: 11848876. 11848876

[19] Cao B, Xu H, Mao C (2011) Transmission electron microscopy as a tool to image bioinorganic nanohybrids: the case of phage-gold nanocomposites. Microsc Res Tech 74: 627-635. doi:10.1002/jemt.21030. PubMed: 21678527. 21678527 PMC3121921

[20] Movasaghi Z, Rehman S, Rehman I (2008) Fourier transform infrared (FTIR) spectroscopy of biological tissues. Appl Spectosc Rev 43: 134-179. doi:10.1080/05704920701829043.

[21] Jena KK, Chattopadhyay DK, Raju K (2007) Synthesis and characterization of hyperbranched polyurethane–urea coatings. Eur Polym J 43: 1825-1837. doi:10.1016/j.eurpolymj.2007.02.042.

[22] Ami D, Natalello A, Doglia SM (2012) Fourier transform infrared microspectroscopy of complex biological systems: from intact cells to whole organisms. Methods Mol Biol 895: 85-100. doi:10.1007/978-1-61779-927-3_7. PubMed: 22760314. 22760314

[23] Nakamoto K (2009) Infrared and raman spectra of inorganic and coordination compounds part A: theory and applications in inorganic chemistry sixth edition. John Wiley & Sons Inc. p. 292.

[24] Bissessur R, Liu PKY, Scully SF (2006) Intercalation of polypyrrole into graphite oxide. Synth Met 156: 1023-1027. doi:10.1016/j.synthmet.2006.06.024.

[25] Hemminga MA, Vos WL, Nazarov PV, Koehorst RB, Stopar D et al. (2010) Viruses: incredible nanomachines: new advances with filamentous phages. Eur Biophys J 39: 541-550. doi:10.1007/s00249-009-0523-0. PubMed: 19680644. 19680644 PMC2841255

[26] Stavis C, Clare TL, Butler JE, Radadia AD, Hamers RJ et al. (2011) Surface functionalization of thin-film diamond for highly stable and selective biological interfaces. Proc of the Natl Acad of Sciences of the USA 108: 983-988. doi:10.1073/pnas.1006660107. PubMed: 20884854. PMC3024699 20884854

[27] Tauson VL, Goettlicher J, Sapozhnikov AN, Mangold S, Lustenberg EE (2012) Sulphur speciation in lazurite-type minerals (Na,Ca)_8_[Al_6_Si_6_O_24_](SO_4_,S)_2_ and their annealing products: a comparative XPS and XAS study. Eur J Mineral 24: 133-152. doi:10.1127/0935-1221/2011/0023-2132.

[28] Dong D, Sutaria S, Hwangbo JY, Chen P (2013) A simple and rapid method to isolate purer M13 phage by isoelectric precipitation. Appl Microbiol Biotechnol 97: 8023-8029. doi:10.1007/s00253-013-5049-9. PubMed: 23807666. 23807666

[29] Su YS, Manthiram A (2012) Lithium-sulphur batteries with a microporous carbon paper as a bifunctional interlayer. Nat Commun 3: 1166. doi:10.1038/ncomms2163. PubMed: 23132016. 23132016

[30] Ever S, Nazar LF (2012) New approaches for high energy density lithium-sulfur battery cathodes. Acc Chem Res. doi:10.1021/ar3001348. 23054430

[31] Seh ZW, Li WY, Cha JJ, Zheng GY , Cui Y et al. (2013) Sulphur–TiO2 yolk–shell nanoarchitecture with internal void space for long-cycle lithium–sulphur batteries. Nat Commun 4: 1331. doi:10.1038/ncomms2327. PubMed: 23299881. 23299881

[32] Ji X, Lee KT, Nazar LF (2009) A highly ordered nanostructured carbon–sulphur cathode for lithium–sulphur batteries. Nat Mater 8: 500-506. doi:10.1038/nmat2460. PubMed: 19448613. 19448613

